# Supplementary material for: A Novel FLCN Variant in a Suspected Birt–Hogg–Dubè Syndrome Patient
Source: Int J Mol Sci. 2023 Aug 4;24(15):12418. doi: 10.3390/ijms241512418 (PMC10419138; doi:10.3390/ijms241512418)
Supplement: Supplementary file 1 [file ijms-24-12418-s001.zip › ijms-2530624-supplementary.pdf]

Table S1. Panel of 32 genes used for NGS analysis through SOPHiA Custom Hereditary Cancer Solution (CHCS).

ABRAXAS1	APC	ATM	BARD1	BRCA1	BRCA2	BRIP1	CDH1	CHEK2	EPCAM
MLH1	MRE11	MSH2	MSH6	MUTYH	NBN	PALB2	PIK3CA	PMS2	PTEN
PIK3CA	RAD50	RAD51C	RAD51D	STK11	TP53	XRCC2	FAM175A	MEN1	CDK4
CDKN2A	RET								

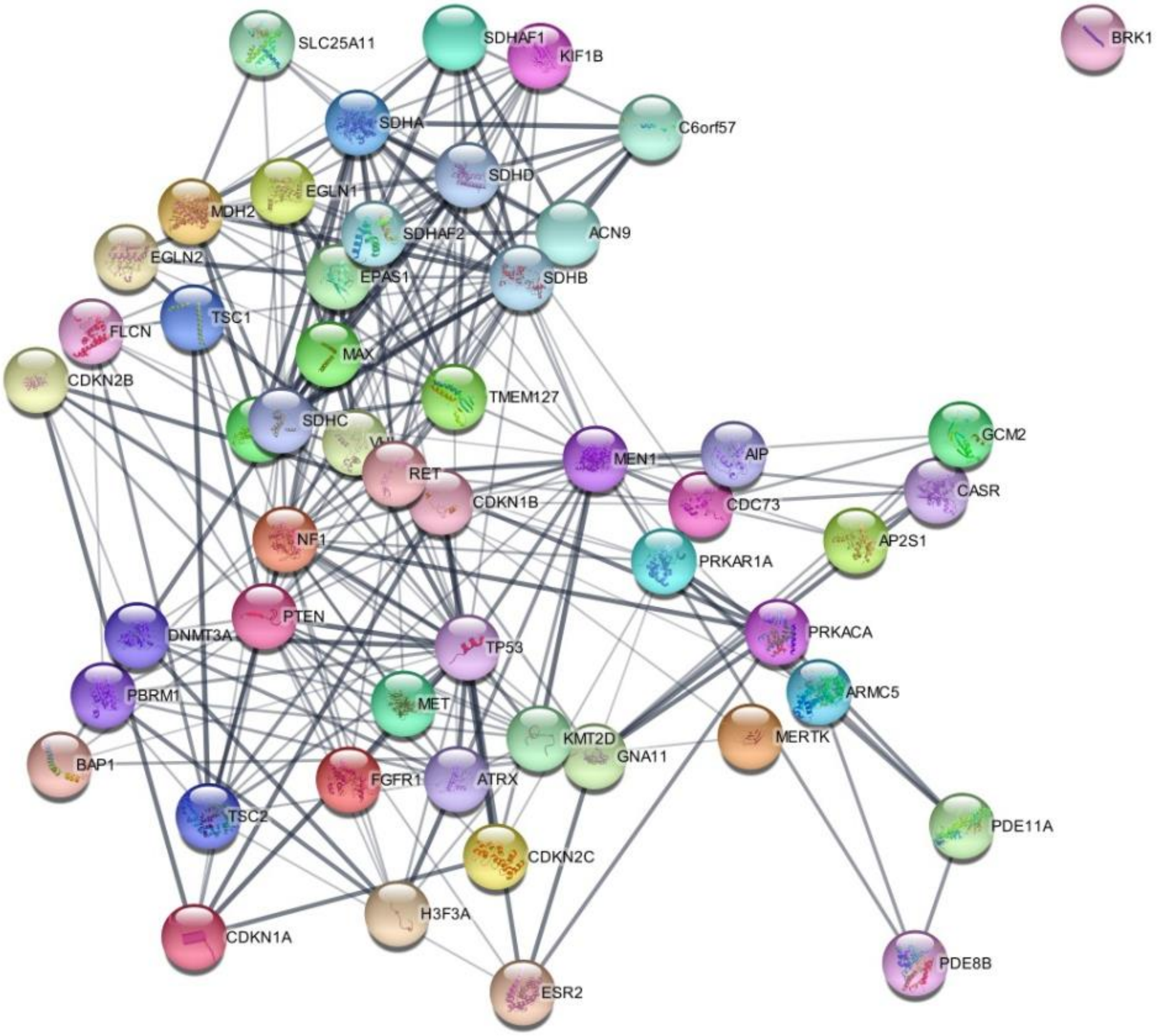

Figure S1. Protein-protein interactions (PPI) network obtained through Cytoscape tool v3.10.0 to define the connections between the proteins encoded by the 52 genes included within the custom panel.
